# Promising Preserving Agents from Sage and Basil: A Case Study with Yogurts

**DOI:** 10.3390/foods10030676

**Published:** 2021-03-22

**Authors:** Jonata M. Ueda, Mariana C. Pedrosa, Filipa A. Fernandes, Paula Rodrigues, Bruno Melgar, Maria Inês Dias, José Pinela, Ricardo C. Calhelha, Marija Ivanov, Marina Soković, Sandrina A. Heleno, Márcio Carocho, Rafael P. Ineu, Isabel C. F. R. Ferreira, Lillian Barros

**Affiliations:** 1 Centro de Investigação de Montanha (CIMO), Instituto Politécnico de Bragança, Campus de Santa Apolónia, 5300-253 Bragança, Portugal; massaoueda@hotmail.com (J.M.U.); marianapedrosa@ipb.pt (M.C.P.); filipaf@ipb.pt (F.A.F.); prodrigues@ipb.pt (P.R.); bruno.melgarc@gmail.com (B.M.); maria.ines@ipb.pt (M.I.D.); jpinela@ipb.pt (J.P.); calhelha@ipb.pt (R.C.C.); mcarocho@ipb.pt (M.C.); iferreira@ipb.pt (I.C.F.R.F.); 2 Campus Campo Mourão (UTFPR-CM), Universidade Tecnológica Federal do Paraná, Campo Mourão 87301-899, Brazil; rafaelineu@gmail.com; 3 Institute for Biological Research “Siniša Stanković”—National Institute of Republic of Serbia, University of Belgrade, Blvd. despot Stefan 142, 11000 Belgrade, Serbia; marija.smiljkovic@ibiss.bg.ac.rs (M.I.); mris@ibiss.bg.ac.rs (M.S.)

**Keywords:** yogurts, natural preservatives, functional foods, sustainable technologies, green solvents

## Abstract

In the present work, sage (*Salvia officinalis* L.) and basil (*Ocimum basilicum* L.) were exploited for their preservative purposes, as viable alternatives to artificial ones. The ultrasound-assisted extraction (UAE) of bioactive compounds was pre-optimized using factorial screening analysis, prior to applying response surface methodology (RSM). The obtained extracts were characterized in terms of phenolic compounds by high-performance liquid chromatography coupled to photodiode array detector and mass spectrometer HPLC-DAD-ESI/MS and bioactivities, namely the antioxidant, antimicrobial and cytotoxic potential. In addition, the most promising extracts were incorporated into yogurts, that were further screened for nutritional and physico-chemical properties and microbial load, over a shelf life of 14 days. According to the obtained results, the solvent percentage is the most relevant factor for obtaining rosmarinic acid-rich extract, followed by the extraction time and ultrasonic power. For the antioxidant and antimicrobial activity, sage showed the best result for both analysis and none of the two plant extracts were hepatotoxic. Finally, both extracts did not show changes in the physicochemical and nutritional characteristics of the yogurts and did not interfere with the growth of lactic acid bacteria, an important microorganism during yogurt fermentation. These results highlight the high potential of sage and basil as natural preservatives.

## 1. Introduction

Foods are susceptible to various forms of degradation, whether due to the oxygen present in the atmosphere, microbiological, enzymatic, physical and other factors. About 25% of all food produced is lost due to microbial contamination along the supply chain and more than 40% of the damage generated to the foods occurs in the retail area and in contact with the consumer, being discarded as waste [1,2].

To overcome these issues, the food industry relies on additives, namely preservatives. These additives, mostly of artificial origin must be approved by regulatory institutions and cannot alter the food beyond the specific purpose for which they are designed [3]. Nevertheless, due to several side effects related with the consumption of these molecules, natural additives arise as a hot topic in food research, allied to the fact that consumers started tending towards natural based diets and consequently natural-based food additives.

The use of natural preservatives covers two important challenges: ensuring consumer safety with the use of natural sources and reduces the amount of waste from degraded foods [2,4,5].

Plants have been studied for their preservative capacity, since compounds produced by their secondary metabolism exhibit strong antioxidant and antimicrobial activity, justified as a form of defense against microorganisms, pests and oxidizing agents [3,6,7].

The plants of the Lamiaceae family have been used for centuries due to their medicinal properties, with more than 250 genera and 7000 species known. As for their classification, these belong to the kingdom Plantae, division Magnoliophyta, class Magnoliopsida and order Lamiales [8].

Basil and sage have been the subject of several studies, showing their beneficial properties to health, due to the bioactive compounds present in plants. The studies are based on its antioxidant and antimicrobial properties, in addition to medicinal effects such as anti-inflammatory, antidiabetic and anticancer properties [9,10,11,12].

Thus, the main objective of the present work was to develop an enriched extract in bioactive molecules, from sage (*Salvia officinalis* L.) and basil (*Ocimum basilicum* L.), namely in terms of antioxidant and antimicrobial agents, to exploit as natural preservatives.

## 2. Materials and Methods

### 2.1. Samples, Standards and Reagents

Samples of dried leaves of sage and basil were obtained from “Cantinho das Aromáticas” from Vila Nova de Gaia (Portugal), a company that commercializes aromatic plants. The dried leaves were ground to obtain a fine powder (approximately 20 mesh) and stored until further analysis. All reagents and chemicals were acquired from scientific retailers with at least analysis purity. For high performance liquid chromatography (HPLC), the purity was of HPLC grade.

### 2.2. Extracts Preparation

The extracts were obtained using the UAE (Sonicator QSonica, CL-33 model, Newton, CT, USA). Briefly, a known amount of dried sample was weighed and put it contact with the solvent EtOH/H_2_O (80:20, *v*/*v*), obtaining a concentration of 25 g/L, varying its proportion. Afterwards, the samples were subjected to the UAE in the conditions defined by the RSM design. The samples were further centrifuged (5000 rpm, 20 min at 10 °C), filtered through a paper filter (Whatman n° 4) and the ethanol was evaporated at 40 °C using a rotated vacuum evaporator (Büchi, R-210, Flawil, Switzerland). Lastly, the aqueous fraction was lyophilized (−47 °C, 0.045 bar, FreeZone 4.5, Labconco, Kansas City, MO, USA).

### 2.3. Screening Analysis Prior to RSM Technique

Based on the information available in literature, sage and basil are rich sources of rosmarinic acid, a strong antioxidant and antimicrobial agent [13,14,15]. Therefore, the optimization procedures were conducted with the aim at obtaining enriched extract in rosmarinic acids, in high yields and purity.

According to the defined optimization protocol, three different variables were optimized, namely the ultrasonic potency, the solvent percentage (varying ethanol:water proportions) and the extraction time. The used solid/liquid ration was fixed in 25 g/L and the extraction conditions were defined by the responses of the different factorial designs employed.

A fractional factorial design was applied, targeting rosmarinic acid, using a normal two-level factorial design with resolution IV (2^(3-1)^) with the Design Expert software (Stat-Ease, Inc., Minneapolis, MN, USA). This model included 4 runs with the fixed variables varying between: X1—time (7.5 to 12.5 min), X2—solvent (0 to 80% ethanol) and X3—ultrasonic potency (275 to 450 watts) and considering the amount of rosmarinic acid (Y1), the dependent variable. The intercept factors function was used with Intercept = Intercept + ABC, A = A + BC, B = B + AC and C = C + AB. Afterwards, through a general factorial design it was possible to narrow down on the optimal conditions, fixing the potency at 55% and solvent percentage at 80, varying time (10 s to 5 min). Finally, the other parameters were fixed and the temperature varied (X4) (20 to 75 °C), establishing the condition to obtain high yield of rosmarinic acid.

### 2.4. Phenolic Profile

The extracts from the different optimization conditions for basil and sage were dissolved in EtOH (20:80, *v*/*v*) at 10 mg/mL and then filtered and injected.

The phenolic compounds were identified through high-performance liquid chromatography coupled to a diode array detector (DAD) set at 280, 330 and 370 nm of wavelength and coupled to a mass detector (MS) [16].

Chromatographic data related to the identification and quantification of phenolic compounds for the optimization of extraction were acquired using a Dionex Ultimate 3000 UPLC (Thermo Scientific, San Jose, CA, USA), in negative mode. Chromatographic separation was performed using a Waters Spherisorb S3 ODS-2 C18 column (3 μm, 4.6 mm × 150 mm, Waters, Milford, MA, USA) at 35 °C.

Phenolic compounds were identified considering the retention time, UV-Vis and mass spectrum, in comparison with commercial standards and data available in the literature. The calibration curves of the phenolic standards were constructed using the UV-Vis signal to obtain quantitative analysis. In the case of unavailable commercial standards, the compounds were quantified by a calibration curve of the most similar compounds available. The results were expresses in mg/g of extract.

### 2.5. Bioactive Properties and Cytotoxicity

#### 2.5.1. Antioxidant Activity

The antioxidant activity was determined by Oxidative Hemolysis Inhibition Assay (OxHLIA) [17]. Briefly, an erythrocyte solution (2.8%, *v*/*v*; 200 µL) prepared in phosphate-buffered saline (PBS, pH 7.4) was mixed with 400 µL of either extract solution (6–500 μg/mL in PBS), PBS (control), water (for complete hemolysis), or the positive control Trolox (7.81–250 µg/mL PBS). After pre-incubation at 37 °C for 10 min with shaking, 200 μL of 2,2′-azobis(2-methylpropionamidine) dihydrochloride (AAPH, 160 mM in PBS, from Sigma-Aldrich, St. Louis, MO, USA) were added and the optical density was measured at 690 nm every ~10 min in a microplate reader (Bio-Tek Instruments, ELX800, Winooski, VT, USA) until complete hemolysis. The Δ*t* values (min) resulting from the half hemolysis time (H*t*_50_ values) obtained from the hemolytic curves of each extract sample concentration minus the H*t*_50_ value of the PBS control were correlated to the respective extract concentration to obtain the IC_50_ values (inhibition concentration, µg/mL), which were calculated for time periods of 120 and 180 min, i.e., extract concentration required to protect 50% of the erythrocyte population from the hemolytic action of AAPH for 120 and 180 min.

#### 2.5.2. Antimicrobial Activity

In order to evaluate the antimicrobial potential of hydroethanolic extracts of sage and basil, the microdilution method was used [18].

For antibacterial activity, the following Gram-positive bacteria were analyzed: *Staphylococcus aureus* (ATCC 11632), *Bacillus cereus* (clinical isolate) and *Listeria monocytogenes* (NCTC 7973), as well as the following Gram-negative bacteria: *Escherichia coli* (ATCC 25922), *Salmonella Typhimurium* (ATCC 13311) and *Enterobacter cloacae* (ATCC 35030).

For antifungal assays, six micromycetes were used: *Aspergillus fumigatus* (human isolate), *Aspergillus niger* (ATCC 6275), *Aspergillus versicolor* (ATCC11730), *Penicillium funiculosum* (ATCC 36839), *Trichoderma viride* (IAM 5061) and *Penicillium verrucosum var. cyclopium* (food isolate).

The results were showed through complete inhibition of microbial growth (MIC, minimum inhibitory concentration), through the colorimetric microbial viability assay, as well as the minimum bactericidal/fungicidal concentration (MBC and MFC, respectively). The positive controls used were artificial preservatives E211 and E224 (sodium benzoate and potassium metabisulfite, respectively) (Sigma-Aldrich, St. Louis, MO, USA).

#### 2.5.3. Evaluation of the Hepatotoxicity

In order to assess the toxicity of the obtained extracts in normal cells and ensure the safety of the active concentrations, the extracts were tested through the sulphorodamine B assay on a cell line obtained from normal porcine liver cells (assigned as PLP2) in a range of concentrations between 400 to 1.56 μg/mL [19].

The results were expressed in GI_50_ values (growth inhibition concentration, that inhibits 50% of cell growth) and ellipticine was used as the positive control.

### 2.6. Preparation of the Yogurts and Incorporation Procedure

For the preparation of yogurts, a 125 g natural yogurt was mixed with 1 L of semi-skimmed milk at approximately 50 °C. The lyophilized plant extracts were incorporated (about 30 mg/kg); this amount was based on the amount of potassium sorbate commonly applied in these products. The formulations were placed in a yogurt maker for 8 h at 25 °C. After preparation, a portion of strawberry fruit pulp (approximately 20 g) was added to a yogurt mold and the yogurt formulations were added on top. Yogurts were made with natural fruit pulp, since natural yogurts without added fruit do not require the addition of preservatives. Thus, the extracts were added in the same amount as potassium sorbate, an artificial additive, an amount that it regulated by the legislation in force, since it is the most used additive for this food matrix [20]. All incorporation steps were performed in aseptic conditions to avoid any contamination. After this process, the yogurts were homogenized and stored in the refrigerator at a temperature of 4–5 °C. All samples were analyzed in triplicate during the 14 days, having been analyzed at time 0 (T0), after 7 days (T7) and 14 days (T14). The shelf life of 14 days was selected based on the average shelf life of homemade yogurts.

### 2.7. Physicochemical Analysis

The developed yogurts were analyzed for their color, pH, water activity and texture. Briefly, for color analysis, a Konica Minolta colorimeter (Chiyoda, Tokyo, Japan) Chroma Meter CR-400 was used, with a D65 illuminant, standard established by the International Lighting Commission (CIE), with 8 mm of aperture and observation angle of 10°. Luminosity (L*) and the chromatic coordinates (a* and b*), in which a* represents the red-green tone and b* the yellow-blue tone.

Regarding pH, a Hanna Instruments HI 902 potentiometer (RI, USA) was used, measuring three points for each sample and the water activity was measured using a Dew Point Water Activity Meter 4TE (Aqua Lab, Cromer, Australia). For texture analysis, the developed formulations were subjected to an analysis on a TA.XT plus texturometer from Stable Micro Systems (Vienna Court, Godalming, United Kingdom). Since yogurts are semi-solid foods, it was opted for an inverted extrusion analysis, allowing to verify the firmness, consistency, cohesiveness and cohesiveness work of the yogurts using the inverted extrusion platform with the 45 mm diameter perspex disc and the 45 mm high cup. The analysis trigger was defined as strength, with a threshold of 5 g and the analysis mode was defined as a distance of 10 mm. The type of test was compression with an initial speed of 5 mm/s, test speed of 3 mm/s and return to origin speed of 10 mm/s.

### 2.8. Nutritional Profile

The analysis of the nutritional profile over the shelf life was made by evaluating the proximate composition, such as the content of fatty acids, soluble sugars and organic acids.

Macronutrients and energetic value: Regarding the proximate composition, the content of crude fat, proteins, moisture and ashes was determined, according to the Official Methods of Analysis, 17th edition [21].

The proteins were determined through the Macro–Kjedahl method, following the AOAC 920.87 methodology, using a conversion factor of 6.38 (dairy products).

The moisture was evaluated using a moisture meter (PMB 163 Moisture Analyzer, Adam Equipment, Oxford), following the AOAC 925.09 methodology.

The ash content was determined according to the AOAC 923.03 methodology. The total carbohydrates were calculated by difference.

Finally, the total energy was calculated according to the following equation: Energy (kcal) = 4 × (g proteins + g carbohydrates) + 9 × (g lipids).

#### 2.8.1. Fatty Acids Profile

Fatty acids were determined by gas chromatography coupled to a flame ionization detector (CG-FID) (DANI 1000, Contone, Switzerland). The procedure begins with the derivatization of the total fat obtained, by adding 5 mL of a 2:1:1 (*v*/*v*/*v*) methanol/sulfuric acid/toluene solution. The samples were then placed in a 50 °C bath with constant agitation for approximately 12 h. The next step was performed with the addition of 3 mL of distilled water and 3 mL of ethyl ether and homogenized in vortex. After phase separation, the supernatant liquid was removed into a vial containing anhydrous sodium sulfate. After this process, the samples were filtered with disposable 0.22 μm nylon filters and injected into the CG-FID, using a Zebron–Kame column (30 m × 0.25 mm × 0.20 μm).

The oven temperature was programmed as follows: initial column temperature at 100 °C, maintained for 2 min, temperature increase from 10 °C/min up to 190 °C and finally, 30 °C/min to 260 °C, maintained for 2 min. The carrier gas (hydrogen) was maintained at 1.1 mL/min (0.61 bar), measured at 100 °C. The split injection (1:50) was performed at 250 °C and the identification of the individual fatty acids was obtained by comparing the retention times of the commercial standards FAME Mix C4-C24 (standard 4788-U, Sigma-Aldrich, St. Louis, MO, USA). The results were presented in relative percentage of each quantified fatty acid.

#### 2.8.2. Organic Acids Profile

Organic acids were detected by high performance liquid chromatography coupled with a photodiode detector (HPLC-DAD) (Shimadzu 20A series, Shimadzu Corporation, Kyoto, Japan). Initially, 1 g of sample was weighed, which was added 25 mL of metaphosphoric acid (4.5%) in an aluminum-covered beaker. The samples were kept under magnetic stirring at room temperature for 20 min. Then, the samples were filtered using a filter paper (Whatman No. 4) into a test tube and finally into a 1.5 mL amber vial with the help of a syringe and 0.22 nylon filter, for HPLC-DAD analysis.

The separation of the compounds was carried out through a C18 reverse phase column (250 mm × 4.6 mm, 5 μm, Phenomenex), thermostated at 35 °C and the detection occurred at the wavelengths of 215 and 245 nm through a diode detector (DAD). The elution solvent used was sulfuric acid (3.6 mM). For the identification and quantification of organic acids, retention time and spectra of commercial standards were compared, as well as their respective calibration curves. The results were presented in g/100 g of fresh weight (fw).

#### 2.8.3. Soluble Sugars

Soluble sugars were determined by HPLC coupled with a refractive index detector (HPLC-RI) (Knauer, Smartline System 1000, Berlin, Germany). About 1 g of the sample was weighed and 1 mL of a standard melezitose solution (IS, 25 mg/mL) and 40 mL of an 80:20 (*v*/*v*) aqueous ethanol solution were added. The samples were in a thermostatic bath at 80 °C for 1 h and 30 min, being stirred every 15 min. After this process, the ethanol was evaporated in the rotary evaporator and the volume was made up to 5 mL with distilled water in volumetric flask. Finally, the samples were filtered with 0.22 μm filters and injected on the HPLC-RI.

For the determination of free sugars, a 100-5 NH_2_ Eurospher column (4.6 × 250 mm, 5 μm, Knauer) was used. The mobile phase used was acetonitrile/deionized water (70:30 *v*/*v*) at 35 °C with a flow rate of 1 mL/min (oven 7971 R Grace, Berlin, Germany). The identification and quantification were performed using the retention times of commercial standards. The data were analysed using Clarity 2.4 software (DataApex, Prague, Czech Republic). Finally, the results were expressed in g/100 g of fresh weight (fw).

### 2.9. Microbiological Analysis

To determine the microbial load in the yogurts along the shelf life of 14 days, the following microorganisms were analyzed: total aerobic mesophiles, coliforms, molds and yeasts and psychrophilic lactic acid bacteria. The sample preparation followed the procedure described in the International Organization for Standardization [22].

These analyses were performed immediately after the yogurt preparation (T0), after 7 days (T7) and after 14 days (T14) of storage at 4–5 °C. Initially, 1 g of the yogurt sample was mixed in 9 mL of peptone water and serial dilutions (10^−1^ to 10^−5^) were made.

Total aerobic mesophiles: the incorporation sowing technique was used: 1 mL of each dilution the sample was placed in a Petri dish and 15 mL of Plate Count Agar (PCA), were added. The procedure was performed in duplicate. The plates were then homogenized and after solidification of the medium, they were incubated at 30 °C in an inverted position for 72 h. Counting was performed on plates containing between 15 and 300 colonies (Limit of Quantification (LOQ) = 1 log (Colony Forming Units) CFU/g) [23].

Coliforms: 1 mL of each dilution the sample was inoculated into a plate and 15 mL of Violet Red Bile Lactose Agar (VRBLA) were added, using the incorporation technique. The plates were homogenized and solidified and further incubated at 37 °C for 48 h in an inverted position. Counting was performed on plates containing 10 to 150 colonies, in duplicate (Limit of Quantification (LOQ) = 1 log (Colony Forming Units) CFU/g) [24].

Molds and yeasts: 0.2 mL of each dilution were placed in Petri dishes containing 15 mL of Dichloran Rose Bengal Chloramphenicol (DRBC), in duplicate, using the spread plate technique. The plates were incubated in the upright position at 25 °C for 5 days, with the counting performed on plates that contained less than 150 colonies (LOQ = 1.7 log CFU/g). Yeasts and molds were counted after 3 and 5 days of incubation, respectively [25,26].

Psycrophilic lactic acid bacteria: 1 mL of each dilution was placed in a Petri dish and 15 mL of De Man, Rogosa and Sharpe (MRS) were added, by incorporation technique. After the medium solidified, an extra medium layer (10 mL) was added in order to promote anaerobic conditions. The plates were incubated in an inverted position at 22 °C for 5 days, with the count being made on plates that contained between 15 and 300 colonies (LOQ = 1 log CFU/g) [27].

### 2.10. Statistical Analysis

For the general factorial design, a comparison was made between the different samples through simple analysis of variance (ANOVA) using F-test, together with the Fisher’s Least Significant Difference test, using the Design expert 12.0.1. (Stat-Ease, Inc. Minneapolis, MN, USA) and Statgraphics Centurion XVI software (StatPoint Technologies, Inc. Warrenton, VA, USA) software.

Values are presented as mean ± standard deviation. Regarding the analysis of the parameters of the yogurts, the values were analyzed using a 2-way ANOVA, with the sum of squares of type III, using the SPSS software, version 25. This model of multivariate linear analysis allows treating the two factors, PT (preservative type) and ST (storage time) independently, allowing us to understand the contribution of each one independently of the other, offering a better understanding of the behavior of the various parameters over time. Throughout the whole manuscript, the level of significance was set to 0.05.

## 3. Results and Discussion

### 3.1. Extraction Optimization

#### 3.1.1. Extraction time Assessment

From the obtained results (Supplementary Material Figure S1), the percentage of solvent was the factor with the greatest influence on the extraction of rosmarinic acid, followed by time and finally, ultrasonic power.

Performing a deeper analysis of the extract yield at different times, this factor became an important to investigate. Thus, a new test was carried out, varying the time between 10 s and 5 min.

Sage was the plant with the highest amount of phenolic compounds, but also the highest concentration of rosmarinic acid.

Although sage showed a higher yield in the shortest time, it is important to note that there does not seem to be any specific trend in the different applied times. Basically, high yields of phenolic compounds differ not only in plants, but also at different times. Therefore, two obvious conclusions can be drawn from these results: the UAE extraction power appears to be greater than expected and further analysis on this subject will be performed, since there are not many studies related to extraction time. Above all, it is an interesting result because in RSM tests based on UAE extractions, the tendency is to use it for long periods, even longer than those tested in this work. Therefore, from a detailed study, reducing the use of energy for the extraction of phenolic compounds could lead to incalculable savings.

#### 3.1.2. Extraction Temperature Assessment

In conjunction with the results obtained from the factorial design with the time dependent variable, an extra multilevel analysis based on the effect of temperature was also performed, acting in an interval between 20 and 75 °C. The main idea of incorporating temperature as a factor was the existence of possible thermolabile phenolic compounds. Therefore, the decision to analyze a common temperature range applied in different extractions seemed interesting in order to maximize the selection of factors for future optimizations. Like the time factor, there is also no evident pattern in the extraction yield of basil; therefore, independent interpretations must be highlighted (Supplementary Material Figure S2).

For sage, temperature increase had a positive effect, obtaining a higher yield with the highest temperature tested. Between the intervals from the lowest to the highest temperature, there was an increase of approximately 15% in the extraction yield of rosmarinic acid, showing a statistically significant different (*p* < 0.05). For basil, the highest yield was at 50 °C. From a deeper analysis of the studied compounds, no specific pattern was detected, as supposed at the beginning, finding no degradation of flavonoids or rosmarinic acid. In addition, the difference in response can be attributed to the arrangement of polyphenols in the food matrix, allowing, in a way, a greater ease of extraction in one plant when compared to another and, consequently, the amount of energy required will vary for each plant, so it a greater focus is needed to be placed on future temperature-related tests.

#### 3.1.3. Phenolic Profile

Based on the phenolic composition and on the screening results, the optimal extraction conditions were determined, being time: 1 min; temperature: 75 °C; and ultrasonic power: 375 Watts. Based on these selected conditions, the most promising plant extracts were analyzed, due to their bioactivity and cytotoxicity, for later incorporation into yogurts. The tentative identification and quantification, as well as the retention times of the phenolic compounds, are described in Table 1, as also the antioxidant and antimicrobial activity of the extracts.

The identification was performed taking into account the molecular ion and fragmentation of the compound, but also the retention time, characteristics of ultraviolet-visible (UV-Vis) and comparison with commercial standards when available. Two phenolic acids and one *O*-glycosilated flavonoids derivative were tentatively identified and quantified (Supplementary Material Figure S3). All the three compounds were previously identified and confirmed by other authors [16,28,29]. Peak 3, rosmarinic acid, was identified accordingly and by comparison with the chromatographic response of the available standard compound. Peak 2, luteolin-*O*-glucuronide, was tentatively identified following the previously reported authors [16]. As for peak 1, 4-hydroxy-7-*O*-(3′hydroxy-4′-*O*-glucosylbenzyl)benzyl, was tentatively identified following the description in a study performed in the leaves of *Ilex glabra* L. Gray [30]. As far as the authors knowledge, this is the first report of this compounds in basil and sage hydroethanolic extracts.

Rosmarinic acid was the only compound identified in both extracts evaluated and with relevant concentration. The phenolic composition of each plant extract corroborates with the results obtained in preliminary tests. The different concentration obtained between this result and the previous ones are justified by the fact that each plant (basil and sage) has different optimal extraction conditions, varying the temperature and time for each extract, as described in the previous sections.

### 3.2. Bioactive Properties and Hepatotoxicity

#### 3.2.1. Antioxidant Activity of the Extracts

Table 1 shows the antioxidant capacity of plant extracts and the positive control (Trolox), determined by the OxHLIA method, for the time periods of 120 and 180 min. The lower the extract concentration needed to inhibit oxidative hemolysis, the greater the antioxidant activity.

Sage showed the best protection for the erythrocyte membrane, by inhibiting 50% of oxidative hemolysis, when compared with Trolox in the time of 120 min (IC_50 (120 min)_ values of 2.6 and 41 μg/mL, respectively). Likewise, in the 180 min period, the IC_50 (180 min)_ values were 8.8 and 63 μg/mL, respectively. On the other hand, basil showed IC_50_ values for 120 and 180 min of 60 and 87 μg/mL, respectively.

In terms of antioxidant activity, sage extract resulted in the highest antioxidant activity, while it is also known for its high antioxidant capacity [29,31,32]. Therefore, it appears that the antioxidant capacity of the plant extracts is possibly related to the phenolic composition, as shown in Section 3.1.3. It is important to note that Trolox is a pure compound, while plant extracts are a mixture of compounds, with or without bioactive properties, which could explain the lowest antioxidant activity of the extracts when compared to Trolox [33].

The antioxidant capacity can be divided into several categories: highly active (IC_50_ < 50 μg/mL); moderately active (50 < IC_50_ < 100 μg/mL); slightly active (100 < IC_50_ < 200 μg/mL) and practically inactive (IC_50_ > 200 μg/mL) [34]. In relation to this classification, sage extract was evaluated with high antioxidant activity and basil extract with moderate antioxidant capacity.

Two basil extracts (*Ocimum basilicum* cv. Cinnamon and *Ocimum × citriodorum*) was explored and extracted from a hydroethanolic solution at room temperature, resulting in IC_50_ values for ‘Cinnamon’ and *Ocimum × citriodorum* of 48 and 54 μg/mL, respectively, for Δ*t* = 60 min [35]. The authors also evaluated the infusion, obtaining values of 27.6 and 26.9 μg/mL. These results corroborate that the extraction method has high interference in the antioxidant activity of the extract.

To the best of the authors knowledge, no studies were found regarding sage performed by the same method to determine antioxidant activity (OxHLIA), however, one study evaluated the antioxidant capacity of sage extract by the TBARS method and found good results (0.457 mg of MDA/kg of sample) [36]. The present study also confirmed this high antioxidant activity (2.6 μg/mL).

#### 3.2.2. Antimicrobial Activity of the Extracts

Table 1 presents the antibacterial activity of the extracts against several Gram negative and positive bacteria, considered food contaminant. Sage showed an inhibitory concentration of 1 mg/mL and bactericidal concentration of 2 mg/mL for all bacteria tested. Basil had a higher inhibitory and bactericidal concentration (2 and 4 mg/mL, respectively) for all bacteria, except for *Bacillus cereus*, which showed and inhibitory and bactericidal concentration equivalent to sage.

Regarding the positive controls E211 and E224 (sodium benzoate and potassium metabisulfite, respectively), all plant extracts obtained superior results in inhibiting *S. aureus* when compared with E211 and superior in inhibiting *B. cereus* when compared with E224. The synthetic additives vary widely in the concentration to inhibit each type of bacteria. For example, E211 requires 4 mg/mL for the inhibition of *S. aureus* and only 0.5 mg/mL for the inhibition of *B. cereus*, indicating that the additive is selective for each species. Plant extracts, on the other hand, inhibit all bacteria at the same rate, with the exception of basil for *B. cereus*, demonstrating the wide application of plant extracts in various food matrices.

Table 1 also shows the antifungal activity of plant extracts, with fungi being more susceptible to the extracts when compared to bacteria. Sage and basil showed similar antifungal activity, with values between 0.25 and 1 mg/mL to achieve their inhibitory and fungicidal capacity.

Comparing the plant extracts with the synthetic preservatives, the extracts were equivalent or superior in inhibiting all tested fungi.

Basil extracts through decoction can obtain for the same bacteria MIC values between 0.125 and 0.250 mg/mL [28]. For fungi, the MIC values vary between 0.062 and 0.250 mg/mL for the same ones tested in this work. Was also verified the antimicrobial activity of basil extracts using maceration with hydroethanolic solution (80:20, *v*/*v*), obtaining a MIC between 0.10 and 0.45 mg/mL for bacteria, whereas for fungi obtained values between 0.20 and 0.60 mg/mL [37]. With the essential oil obtained from hydrodistillation, the authors analyzed the antimicrobial action of basil extracts, obtaining an MIC for several bacteria between 0.25 and 1 mg/mL [38].

Therefore, it appears that the extraction method significantly affects the antimicrobial activity of the extracts, as well as the antioxidant activity.

#### 3.2.3. Hepatotoxicity of the Extracts

Performing toxicity assessment is extremely important, since toxic compounds can generate the production of several metabolites, leading to toxicity [19]. As for cytotoxic activity, both plant extracts did not show hepatotoxicity in PLP2 cells at the maximum tested concentration of 400 μg/mL.

Basil extracts using the decoction method showed no hepatotoxicity in the tested extracts (GI_50_ > 400 μg/mL) [28]. The cytotoxicity of *Salvia officinalis Icterina* extracted by decoction presented GI_50_ = 304.9 μg/mL, according to the authors [39]. Although the extraction methods differ between the author, none of the studies presented hepatotoxicity between the extracts, regardless of the extraction techniques.

### 3.3. Physicochemical Analysis of the Yogurts

Table 2 indicates the physicochemical parameters of the yogurts, such as the three-color coordinates (L*, a* and b*), water activity, pH and texture characteristics. The table representation follows an analysis of the two factors, storage time (ST) and preservative type (PT), in an independent way to provide a better understanding of the effects of each factor on the results presented, or, if possible, there was an interaction between both is present. Thus, the upper part of the table represents the passage of time but included on each day are all different types of yogurts and at the bottom, for each type of preservative extract, the three analysis times are included. When each of the factor can be analyzed independently (*p*-value ST × PT > 0.05), the classification is made using post-hoc testes (Tukey’s test for homoscedastic samples and Tahmane T2 for non-homoscedastic ones); however, when *p*-value TP × TC < 0.05, then, no classification can be performed and thus, for some cases, only general trends can be obtained through the estimated marginal means plots (EMM).

For the color analysis (Supplementary Material Figure S4), among the used preservatives, only for b* (yellow-blue) was there a significant interaction among the two factors. Regarding storage time, there were statistically significant differences (*p* < 0.05) sought for color, in which the L* parameter differed between days 0 and 7, becoming slightly lighter with the passage of time and a* (green–red) between days 7 and 14, registering a tendency to reduce the red color. Therefore, it appears that the storage time had more influence on the color of the yogurts than the type of extracts.

Regarding the water activity, from the *p*-value it is possible to verify that both factors did not reveal a significant interaction and both could be classified independently. Thus, it is noticed that over the 14 days the water activity increases, very slightly and that all preservative extracts, as well as potassium sorbate, also decrease this parameter, with significant differences when compared to the control. Finally, for the pH value, during the 14 days there were no significant differences, not even with the addition of the different extracts. This result is quite satisfactory, since changes in the pH of foods are generally related to changes in texture or, above all, organoleptic characteristics, which should be avoided when searching for new food additives.

Although the authors did not obtain the same results for color parameters, the samples with addition of preservatives (natural and synthetic) did not differ from the control, as expected from a food additive [40]. The addition of fruit pulp to the yogurts made in this work justifies the difference in the result of the color parameters obtained by the previously authors. Nevertheless, the samples also tended to reduce the reddish tone over time, as verified by the authors.

The texture profile of the yogurts at different times of analysis was performed using the inverted extrusion test in order to obtain the following parameters: hardness, consistency, cohesiveness and work of cohesiveness.

Thus, from Table 2 it can be understood that both factors, ST and PT had a significant interaction on the slight changes undergone in terms of texture over the 14 days, revealing that the use of plant extracts does not interfere with the rheological properties of yogurts.

### 3.4. Nutritional Profile

#### 3.4.1. Macronutrients and Energetic Value

Table 3 shows the composition of yogurts and their assessment over the storage time (0, 7 and 14 days), as well as the effect of the preservatives used (basil, sage and potassium sorbate) when compared to control.

As for nutritional composition, moisture was the most abundant parameter, as expected due to the high amount of water present in yogurts. The average moisture (87.75 g/100 g) was close to that reported by the authors (84.79 g/100 g and 87.47 g/100 g, respectively) [40,41]. After moisture, the most abundant components were carbohydrates and proteins, in which carbohydrates had an average of 7.4 g/100 g and 2.88 g/100 g for proteins. The other authors obtained 5.36 and 5.55 g/100 g for carbohydrates and 5.62 and 3.77 g/100 g for proteins, respectively [40,41]. Finally, crude fat was the nutrient found in the least abundance. Only for carbohydrates was there a significant interaction revealing that the value of this nutrient changed due to storage time and the incorporation of extracts. There were no significant differences in moisture over the 14 days of storage time or with the incorporation of different plant extracts or potassium sorbate, showing that none of them had an influence on this nutrient. For crude fat, the variation occurred mainly over time, with a significant difference from 0 to 14 days, with the same difference sought for ashes. The energy contribution of yogurts was not affected by the addition of different preservatives. Effectively, the addition of the different extraction did not have any concrete action on yogurts, which is a positive fact, since food additives cannot have any type of nutritional contribution of change the nutritional profile of the foods in which they are added in any way. The storage time (ST) has a greater influence on samples than the type of preservative used (PT), also reported by other authors [40].

#### 3.4.2. Fatty Acids

Table 4 presents the amounts of the most abundant fatty acids found in the yogurts, expressed as a relative percentage. Of the 13 identified fatty acids, saturated ones (SFA) proved to be the most abundant group, both in quantity and in number of individual molecules, reaching 78%, leaving only about 20% for monounsaturated fatty acids. Regarding polyunsaturated fatty acids, only linoleic acid (C18:2) was recorded with a percentage that varied between 1 and 2%. This proportion was also reported by other authors, in which SFA > MUFA > PUFA [41].

Palmitic (C16:0), oleic (C18:1), myristic (C14:0) and stearic acids (C18:0) were the most abundant individual fatty acids, reaching 38, 18, 14.5 and 12%, respectively. Other studies also identified and quantified the same four fatty acids, as being the most abundant in yogurt samples [40,41,42,43]. Regarding the influence of ST and PT, for all cases there was a significant interaction, showing that plant extracts and potassium sorbate did not influence the change in fatty acids during the 14 days. On the other hand, the shelf life of 14 days does not seem to have been enough to create changes in the fatty acid profile, or the addition of fruit pulp may have had a protective effect against unsaturated fatty acids.

#### 3.4.3. Organic Acids and Soluble Sugars

Table 5 shows the different organic acids and soluble sugars identified in the yogurt samples. For organic acids, among them, the most abundant are lactic and citric acid, which together with the other, oxalic, malic and fumaric, totaling approximately 0.6 g/100 g of fresh yogurt.

Only lactic acid did not reveal a significant interaction, since all other acids had little variation in terms of the passage of time and preservative used, except for oxalic acid, which showed a tendency to increase over time, with statistically significant differences between days 7 and 14. Lactic acid represents an important agent in dairy products, since it is a compound considered as GRAS and used frequently in the food industry as an acidifying additive, preservative and others. Fermentation is defined by the conversion of the substrate by a microorganism, plant or animal cell or enzymes, into a given product. Therefore, despite its importance, lactic acid is not found naturally in food products, but is produced during fermentation by lactic acid bacteria [44,45]. In this case, its increase over time should correspond to the decrease in lactose (soluble sugar) due to fermentation by lactic acid bacteria. The high concentration of citric acid can be justified by the addition of fruit pulp to yogurts. However, several other studies also point out the presence of citric acid in yogurts [46,47,48,49].

As for soluble sugars, four were detected, namely fructose, glucose, sucrose and the most abundant sugar in dairy products, lactose, which registered values between 4 and 5 g/100 g. In terms of classifying the effect of plants extracts and storage time, fructose, glucose and total soluble sugars showed significant interactions, now allowing conclusion to be drawn or saying whether the ST or PT had more influence than the other. Sucrose registered a non-significant interaction but did not show significant changes between each day or extracts incorporated. Only for lactose was it possible to define that the storage time has a greater influence on the amount of lactose than the incorporation of the extracts, registering a statistically significant decrease from day 7 to 14, constant over time. This behavior is corroborated by the constant and significant increase in lactic acid, revealing that lactic acid bacteria were, over time, converting lactose into lactic acid and that plant extracts did not change this natural phenomenon in yogurts. During lactic fermentation, lactose is not fully converted, remaining at levels up to 4 g/100 g of product [45]. On other studies was found an average of 4.17 g/100 g of lactose (without adding pulp) and 3.8 g/100 g (with added pulp) [41,43]. The difference in the results obtained can be justified by the different way of producing the yogurt of the brand of the yogurt used, such as the addition or not of fruit pulp.

### 3.5. Microbiological Analysis

**Figure 1:**
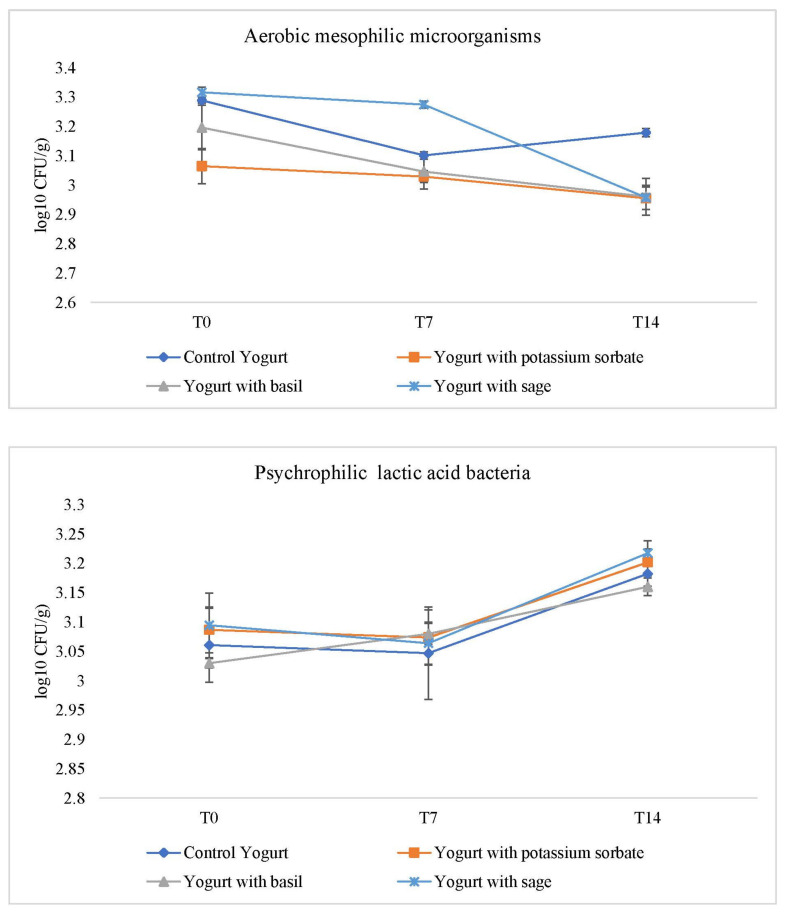
Aerobic mesophilic (top) and psychrophilic lactic acid bacteria (bottom) microbial load over 14 days.

Figure 1 shows the counts for aerobic mesophilic microorganisms and psychrophilic lactic acid bacteria, respectively. Coliforms, molds and yeasts were not detected over the 14 days evaluated.

For mesophilic aerobic microorganisms, statistically significant differences (Data not shown), were verified from T0 to T14, showed by the decrease in these microorganisms in the formulations with sage, basil and potassium sorbate, highlighting that basil and sage presented the same behavior of potassium sorbate. Regarding the control yogurt, statistical significant differences were observed from T7 to T14, by the increase in these microorganisms, due to the absence of a preservative agent capable of inhibiting their growth. Comparing the different treatments, in T7 statistically significant differences were verified from sage to the rest of the formulations, due to the higher counts of these microorganisms. Nevertheless, in T14 sage, basil and potassium sorbate presented no statistically significant differences among each other, showing lower values when compared with the control yogurt. These data suggest that sage’s inhibition of microbial growth started later (7 days), but at T14 showed similar values with no statistical differences when compared to the remaining agents.

Concerning the psychrophilic lactic acid bacteria, the formulations incorporated with potassium sorbate revealed statistically significant differences (data not shown) from T7 to T14, while the formulations with basil showed statistically significative differences from T0 to T7 and also from T7 to T14, verified by the increase in these bacteria. Sage incorporation caused less effects on the growth of these bacteria, when compared to potassium sorbate and basil. These data are important because these bacteria are essential in these products and a negative interference is prejudicial to the yogurt development.

There is a slight increase in the counting of lactic acid bacteria, which is justified by the continued lactic fermentation, as can be confirmed by the reduction of lactose and increase in lactic acid (Table 5; organic acids and soluble sugars).

Lactic acid bacteria are studied for their beneficial probiotic properties, such as improving the function of the epithelial barrier and restoring microbial homeostasis through interactions between microorganisms, in addition to contributing to the functions of the immune system and the elimination of pathogens [50].

## 4. Conclusions

After the optimization of sage and basil extracts it can be concluded that there is no general standard for the time applied and the temperature used, indicating that a longer extraction time and a higher temperature will not necessarily obtain the highest yield of rosmarinic acid in the extracts, while representing a more expensive protocol.

As for the bioactive properties, sage showed the best results, for antioxidant activity, while both extracts (sage and basil) showed similar antimicrobial activity. None of the two plant extracts showed hepatotoxicity in PLP2 cells, at the maximum concentration tested.

It was also verified that among the physicochemical and nutritional parameters, the storage time had a greater influence than the type of preservative used. It is considered a positive point because preserving additives must avoid altering food characteristics.

One of the most relevant studies for the application of preservatives in yogurts is the evaluation of the inhibition of lactic acid bacteria over the shelf life. According to the soluble sugar’s analysis, organic acid and lactic bacteria counting, it was possible to indicate the presence of lactic acid bacteria, justified by the consumption of lactose and production of lactic acid. Therefore, both sage and basil can be promising sources of preservatives in yogurts.

For further investigation, it will be important to test longer times to optimize the yield of the bioactive compounds, as well as a higher concentration of the extracts. It is also necessary to test longer periods of shelf life to check if the preservative is really effective, in addition to testing yogurts with and without fruit pulp, to investigate whether the pulp was also responsible for the preservation of the yogurts (although it does not contain preservatives in its composition) and to check whether there are changes in physical and nutritional characteristics.

## Figures and Tables

**Table 1:** Phenolic profile, antioxidant (μg/mL) and antimicrobial activity (mg/mL) of the plant extracts.

Phenolic Profile									
Peak	Identified Compound					Quantification (mg/g Extract)			
						Basil		Sage	
1 ^a^	4-Hydroxy-7-*O*-(3′hydroxy-4′-*O*-glucosylbenzyl)benzyl					1.00 ± 0.05		n.d	
2 ^b^	Luteolin-*O*-glucuronide					n.d		12.77 ± 0.07	
3 ^c^	Rosmarinic acid					17.90 ± 0.37		23.42 ± 0.26	
	Total					18.90 ± 0.41		36.19 ± 0.33	
Antioxidant activity									
						Δ*t* 120 min		Δ*t* 180 min	
OxHLIA (IC_50_ values)			Basil			60 ± 1		87 ± 1	
			Sage			2.6 ± 0.2		8.8 ± 0.4	
			Trolox			41 ± 1		63 ± 1	
Antimicrobial activity									
		Sage		Basil		E211		E224	
		MIC	MBC	MIC	MBC	MIC	MBC	MIC	MBC
Antibacterial	*Staphylococcus aureus*	1	2	2	4	4	4	1	1
	*Bacillus cereus*	1	2	1	2	0.5	0.5	2	4
	*Listeria monocytogenes*	1	2	2	4	1	2	0.5	1
	*Escherichia coli*	1	2	2	4	1	2	0.5	1
	*Salmonella* *Typhimurium*	1	2	2	4	1	2	1	1
	*Enterobacter cloacae*	1	2	2	4	2	4	0.5	0.5
		MIC	MFC	MIC	MFC	MIC	MFC	MIC	MFC
Antifungal	*Aspergillus fumigatus*	0.25	0.5	0.25	0.5	1	2	1	1
	*Aspergillus niger*	0.5	1	0.5	1	1	2	1	1
	*Aspergillus versicolor*	0.25	0.5	0.5	1	2	2	1	1
	*Penicillium funiculosum*	0.25	0.5	0.25	0.5	1	2	0.5	0.5
	*Penicillium verrucosum*	0.25	0.5	0.25	0.5	2	4	1	1
	*Trichoderma viride*	0.5	1	0.5	1	1	2	0.5	0.5

n.d—non detected. Calibration curve standards: ^a^ cinnamic acid (*y* = 1E + 06*x*—222,204, *R*^2^ = 0.9993, LOD = 0.12 µg/mL and LOQ = 0.83 µg/mL); b—quercetin-3-*O*-glucoside (*y* = 34843x—160173; *R*^2^ = 1.000; LOD = 0.21 µg/mL and LOQ = 0.71 µg/mL); c—rosmarinic acid (*y* = 191291*x*—652903; *R*^2^ = 0.999; LOD = 0.15 µg/mL and LOQ = 0.68 µg/mL).

**Table 2:** Physicochemical parameters of the yogurts incorporated with different extracts.

		L*	a*	b*	Water Activity	pH	Hardness (g)	Consistency (g/s)	Cohesiveness (g)	Cohesiveness Work (g/s)
Storage Time (ST)	0 Days	71 ± 3 ^a^	2.6 ± 0.2 ^b^	10.9 ± 0.7	0.992 ± 0.001 ^a^	4.8 ± 0.2	49 ± 8	138 ± 21	−31 ± 7	−22 ± 6
	7 Days	79 ± 2 ^b^	2.8 ± 0.5 ^b^	12 ± 1	0.995 ± 0.001 ^b^	4.8 ± 0.2	56 ± 11	151 ± 26	−38 ± 9	−28 ± 7
	14 Days	82 ± 3 ^b^	2.1 ± 0.2 ^a^	11 ± 2	0.996 ± 0.001 ^b^	4.9 ± 0.1	57 ± 10	146 ± 26	−35 ± 9	−26 ± 8
*p-*value (*n* = 15)	Tukey’s HSD test	<0.001	<0.001	<0.001	<0.001	0.12	0.007	0.075	0.003	0.001
Preservative Type (PT)	Control	78 ± 6	2.7 ± 0.5 ^a^	11.5 ± 0.8	0.997 ± 0.001 ^b^	4.8 ± 0.2	51 ± 8	140 ± 15	−32 ± 6	−24 ± 6
	Basil	76 ± 4	2.5 ± 0.6 ^a^	12 ± 2	0.995 ± 0.001 ^a^	4.9 ± 0.2	53 ± 3	133 ± 9	−30 ± 3	−21 ± 3
	Sage	78 ± 5	2.3 ± 0.3 ^a^	11 ± 1	0.995 ± 0.001 ^a^	4.8 ± 0.2	57 ± 2	152 ± 18	−36 ± 7	−28 ± 5
	Potassium sorbate	77 ± 6	2.0 ± 0.3 ^a^	10 ± 1	0.995 ± 0.001 ^a^	4.91 ± 0.05	42 ± 3	126 ± 14	−28 ± 6	−20 ± 5
*p-*value (*n* = 9)	Tukey’s HSD test	0.235	0.031	<0.001	<0.001	0.162	<0.001	<0.001	<0.001	<0.001
ST×PT (*n* = 45)	*p*-value	0.416	0.096	<0.001	0.852	0.399	0.037	0.027	0.012	0.004

In each row, different letters mean significant statistical differences, with an overall significance value of 0.05. The presented standard deviations were calculated from results obtained under different operational conditions. Therefore, these values should not be regarded as a measure of precision, rather as the range of the recorded values.

**Table 3:** Nutritional composition of the yogurts with the different extracts and preservatives (g/100 g fw).

		Moisture	Crude Fat	Ashes	Protein	Carbohydrates	Energy kcal	Energy kJ
Storage Time (ST)	0 Days	87 ± 2	1.7 ± 0.3 ^b^	1.0 ± 0.2 ^b^	2.9 ± 0.3	8 ± 1	34 ± 7	144 ± 30
	7 Days	88 ± 3	1.2 ± 0.3 ^a^	0.6 ± 0.2 ^a^	3.0 ± 0.7	8 ± 1	31 ± 7	130 ± 27
	14 Days	88 ± 2	1.3 ± 0.5 ^a,b^	0.7 ± 0.2 ^a^	2.7 ± 0.8	7 ± 1	29 ± 6	137 ± 27
*p-*value (*n* = 15)	Tukey’s HSD test	0.088	0.039	<0.001	0.153	0.079	0.068	0.068
Preservative Type (PT)	Control	87 ± 2	1.6 ± 0.6 ^a^	0.8 ± 0.4	2.8 ± 0.4	7 ± 1	33 ± 6	137 ± 27
	Basil	88 ± 2	1.1 ± 0.6 ^a^	0.8 ± 0.1	2.9 ± 0.6	7 ± 1	29 ± 6	120 ± 26
	Sage	89 ± 1	1.1 ± 0.4 ^a^	0.9 ± 0.3	2.9 ± 0.3	8 ± 1	29 ± 4	124 ± 18
	Potassium sorbate	88 ± 2	1.6 ± 0.6 ^a^	0.8 ± 0.3	2.7 ± 0.8	7 ± 2	32 ± 10	136 ± 41
*p-*value (*n* = 9)	Tukey’s HSD test	0.592	0.043	0.725	0.473	0.120	0.207	0.207
ST×PT (*n* = 45)	*p-*value	0.088	0.186	0.306	0.08	0.009	0.11	0.11

In each row, different letters mean significant statistical differences, with an overall significance value of 0.05. The presented standard deviations were calculated from results obtained under different operational conditions. Therefore, these values should not be regarded as a measure of precision, rather as the range of the recorded values.

**Table 4:** Individual fatty acids profile, as well as total saturated fatty acids (SFA) and monounsaturated fatty acids (MUFA) found in the yogurts, expressed as relative percentage.

		C6:0	C8:0	C10:0	C12:0	C14:0	C14:1	C15:0	C16:0	C16:1	C17:0	C18:0	C18:1	C18:2	SFA	MUFA
Storage Time (ST)	0 Days	3.0 ± 0.7	2.1 ± 0.3	4.3 ± 0.7	4.6 ± 0.8	13 ± 1	0.8 ± 0.2	1.22 ± 0.05	37 ± 2	1.3 ± 0.2	0.9 ± 0.3	12 ± 1	17 ± 1	2 ± 1	78 ± 2	20 ± 1
	7 Days	3.2 ± 0.5	1.9 ± 0.3	3.7 ± 0.7	4.1 ± 0.6	12 ± 1	0.7 ± 0.2	1.19 ± 0.05	38 ± 1	1.4 ± 0.2	1.0 ± 0.2	12.7 ± 0.9	18 ± 1	1.5 ± 0.8	78 ± 2	20 ± 1
	14 Days	3.2 ± 0.3	2.1 ± 0.1	4.6 ± 0.3	5.2 ± 0.4	14.5 ± 0.9	0.83 ± 0.09	1.36 ± 0.04	37.7 ± 0.7	1.3 ± 0.1	0.85 ± 0.09	10.8 ± 0.7	16.5 ± 0.5	1.0 ± 0.2	80.4 ± 0.5	18.6 ± 0.6
*p-*value (*n* = 15)	Tukey’s HSD test	0.289	<0.001	<0.001	<0.001	<0.001	0.051	<0.001	0.003	0.045	0.043	<0.001	<0.001	<0.001	<0.001	<0.001
Preservative Type (PT)	Control	2.9 ± 0.6	2.0 ± 0.2	4.2 ± 0.7	4.7 ± 0.7	14 ± 1	0.8 ± 0.2	1.3 ± 0.1	38 ± 1	1.3 ± 0.2	0.8 ± 0.1	12 ± 2	18 ± 1	1.2 ± 0.4	79 ± 1	19 ± 1
	Basil	2.4 ± 0.2	1.9 ± 0.5	4 ± 1	4 ± 1	13 ± 1	0.9 ± 0.2	1.27 ± 0.07	37 ± 2	1.3 ± 0.1	0.81 ± 0.09	12 ± 1	18 ± 2	1.6 ± 0.7	78 ± 3	20 ± 2
	Sage	3.0 ± 0.4	2.1 ± 0.1	4.1 ± 0.4	4.4 ± 0.5	13 ± 1	0.9 ± 0.2	1.2 ± 0.1	38 ± 1	1.4 ± 0.2	1.1 ± 0.3	12 ± 1	17.3 ± 0.7	2 ± 1	78 ± 2	19.5 ± 0.9
	Potassium sorbate	3.4 ± 0.3	2.1 ± 0.4	4.4 ± 0.7	4.9 ± 0.7	14 ± 1	0.8 ± 0.1	1.28 ± 0.06	38 ± 1	1.2 ± 0.2	1.0 ± 0.1	12 ± 2	16.2 ± 0.8	1.0 ± 0.4	80.8 ± 0.7	18 ± 1
*p-*value (*n* = 9)	Tukey’s HSD test	0.037	0.065	0.149	0.022	<0.001	0.078	<0.001	0.025	0.02	<0.001	0.993	<0.001	<0.001	<0.001	0.416
ST×PT (*n* = 45)	*p-*value	<0.001	<0.001	<0.001	<0.001	<0.001	<0.001	0.001	<0.001	<0.001	0.003	<0.001	<0.001	<0.001	<0.001	<0.001

The presented standard deviations were calculated from results obtained under different operational conditions. Therefore, these values should not be regarded as a measure of precision, rather as the range of the recorded values. C6:0—caproic acid; C8:0—caprylic acid; C10:0—capric acid; C12:0—lauric acid; C14:0—myristic acid; C14:1—myristoleic acid; C15:0—pentadecanoic acid; C16:0—palmitic acid; C16:1—palmitoleic acid; C17:0—heptadecanoic acid; C18:0—stearic acid; C18:1—oleic acid; C18:2—linoleic acid; SFA—saturated fatty acids; MUFA—monounsaturated fatty acids.

**Table 5:** Organic acids and soluble sugars profile of yogurts presented in g/100 g fw.

		Oxalic Acid	Malic Acid	Lactic ACID	Citric Acid	Fumaric Acid	Total Organic Acids	Fructose	Glucose	Sucrose	Lactose	Total Soluble Sugars
Storage Time (ST)	0 Days	0.007 ± 0.003	0.13 ± 0.03	0.28 ± 0.08 ^a^	0.22 ± 0.06	0.006 ± 0.003	0.6 ± 0.1	1.9 ± 0.3	0.8 ± 0.1	0.15 ± 0.05	5.1 ± 0.7 ^b^	8 ± 1
	7 Days	0.004 ± 0.003	0.13 ± 0.03	0.30 ± 0.01 ^a^	0.23 ± 0.05	0.007 ± 0.003	0.7 ± 0.1	1.7 ± 0.4	0.7 ± 0.2	0.16 ± 0.08	4.4 ± 0.3 ^a,b^	7 ± 1
	14 Days	0.001 ± 0.001	0.12 ± 0.02	0.37 ± 0.01 ^b^	0.18 ± 0.05	0.004 ± 0.004	0.6 ± 0.1	1.5 ± 0.6	0.6 ± 0.2	0.13 ± 0.09	4.0 ± 0.1 ^a^	6 ± 2
*p-*value (*n* = 15)	Tukey’s HSD test	<0.001	0.238	0.028	0.001	0.036	0.02	0.031	0.003	0.517	0.039	0.029
Preservative Type (PT)	Control	0.005 ± 0.004	0.12 ± 0.02	0.3 ± 0.1	0.19 ± 0.06	0.007 ± 0.003	0.6 ± 0.2	1.7 ± 0.5	0.7 ± 0.2	0.12 ± 0.08	4 ± 1	7 ± 2
	Basil	0.003 ± 0.004	0.11 ± 0.03	0.3 ± 0.1	0.20 ± 0.05	0.005 ± 0.003	0.6 ± 0.2	1.6 ± 0.3	0.7 ± 0.1	0.15 ± 0.08	4.4 ± 0.6	6.9 ± 0.9
	Sage	0.004 ± 0.003	0.13 ± 0.02	0.33 ± 0.08	0.22 ± 0.03	0.003 ± 0.001	0.7 ± 0.1	1.7 ± 0.5	0.7 ± 0.2	0.12 ± 0.07	4 ± 1	7 ± 2
	Potassium sorbate	0.003 ± 0.003	0.13 ± 0.04	0.3 ± 0.1	0.23 ± 0.07	0.009 ± 0.003	0.6 ± 0.2	1.7 ± 0.5	0.7 ± 0.2	0.13 ± 0.04	4 ± 1	7 ± 2
*p-*value (*n* = 9)	Tukey’s HSD test	0.119	0.108	0.705	0.143	0.001	0.532	0.812	0.583	0.135	0.526	0.583
ST × PT (*n* = 45)	*p-*value	0.003	<0.001	0.502	<0.001	0.026	0.037	0.025	0.041	0.112	0.081	0.037

In each row, different letters mean significant statistical differences, with an overall significance value of 0.05. The presented standard deviations were calculated from results obtained under different operational conditions. Therefore, these values should not be regarded as a measure of precision, rather as the range of the recorded values.

## Data Availability

Data is contained within the article or supplementary material.

## Supplementary Materials

The following are available online at https://www.mdpi.com/2304-8158/10/3/676/s1, Figure S1. Pareto diagram of the tested effects and their magnitudes. Figure S2. Effect of temperature on rosmarinic acid yield in the evaluated plants. Figure S3. Phenolic profile of basil extract at 280 nm (top) and sage extract at 280 nm (middle) and 370 nm (bottom). The numbers shown corresponds to the compounds described in Table 1. Figure S4. Color representation of the different yogurts over the 14 days with data collected from the colorimeter.
