# Supplementary material for: Promising Preserving Agents from Sage and Basil: A Case Study with Yogurts
Source: Foods. 2021 Mar 22;10(3):676. doi: 10.3390/foods10030676 (PMC8004741; doi:10.3390/foods10030676)
Supplement: Supplementary file 1 [file foods-10-00676-s001.pdf]

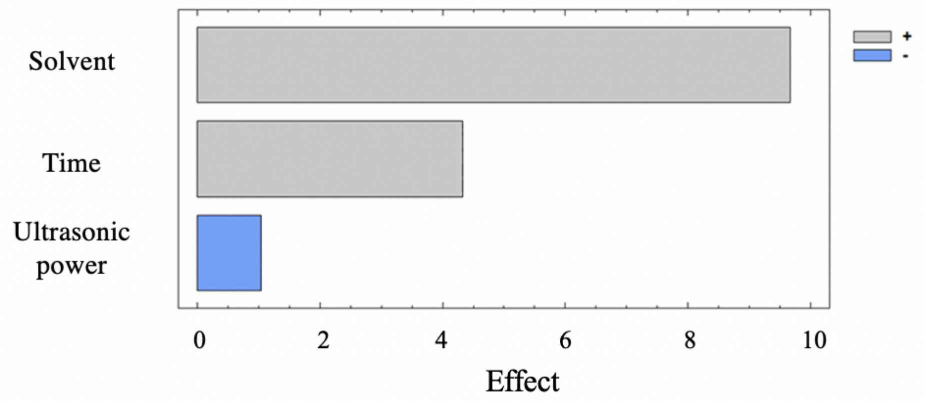

Figure S1. Pareto diagram of the tested effects and their magnitudes.

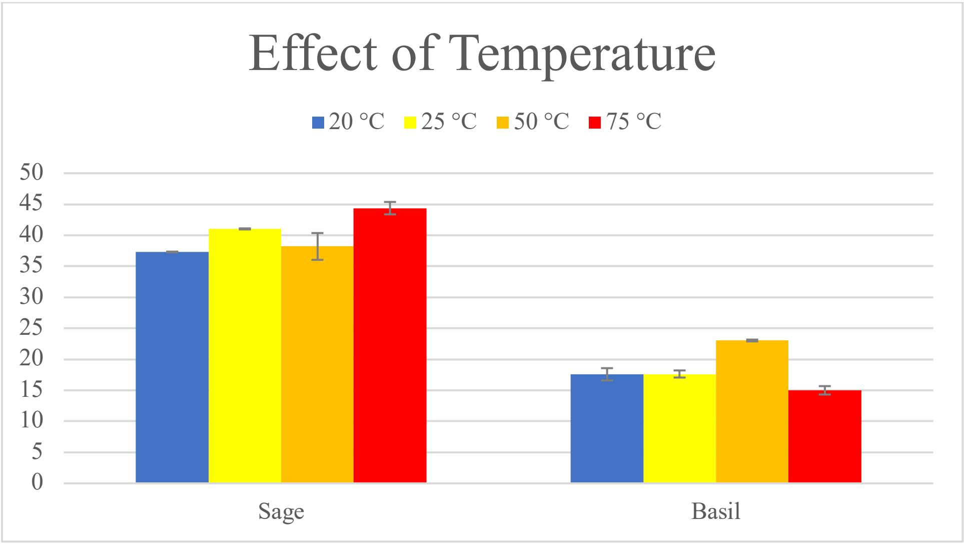

Figure S2. Effect of temperature on rosmarinic acid yield in the evaluated plants.

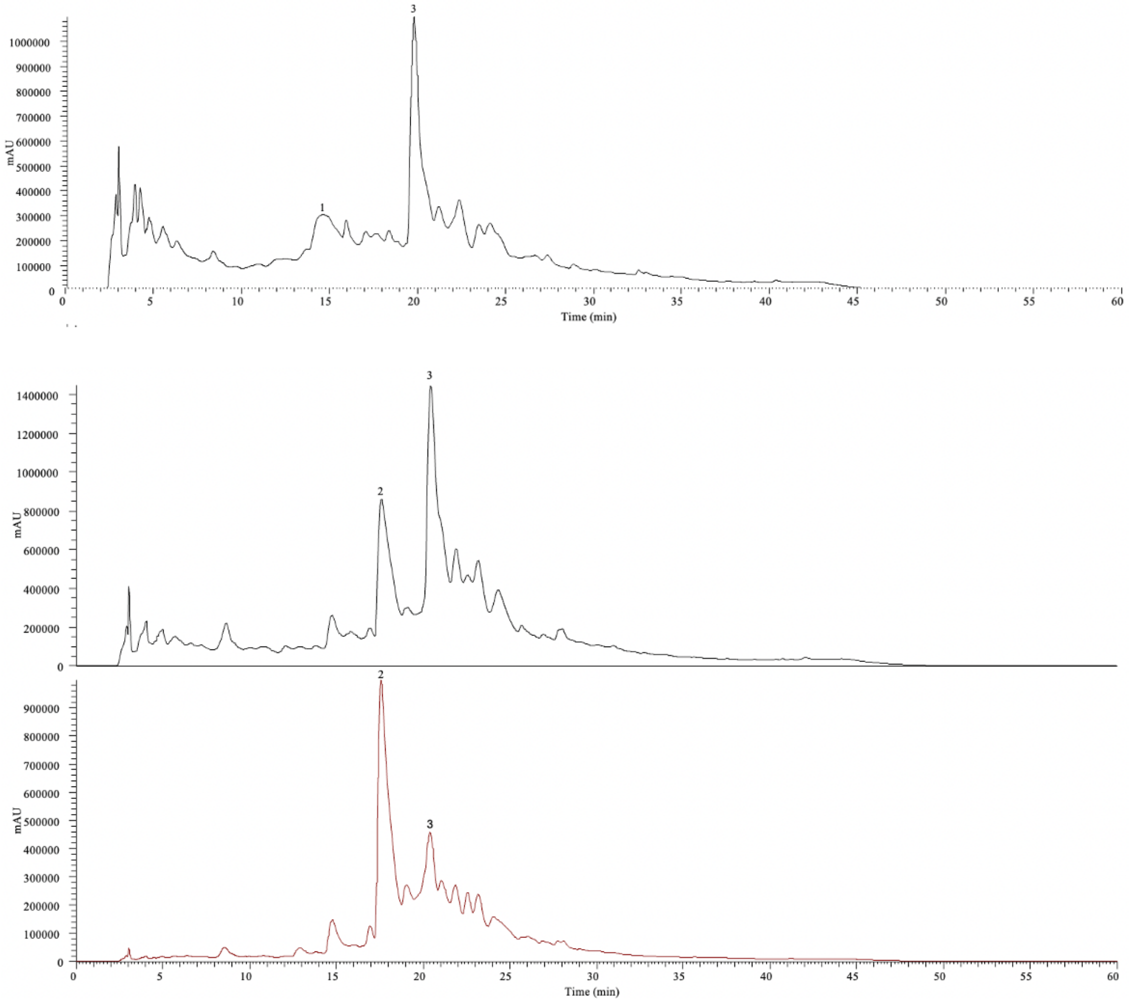

Figure S3. Phenolic profile of basil extract at 280 nm (top) and sage extract at 280 nm (middle) and 370 nm (bottom). The numbers shown corresponds to the compounds described in **Erro! A origem da referência não foi encontrada.**.

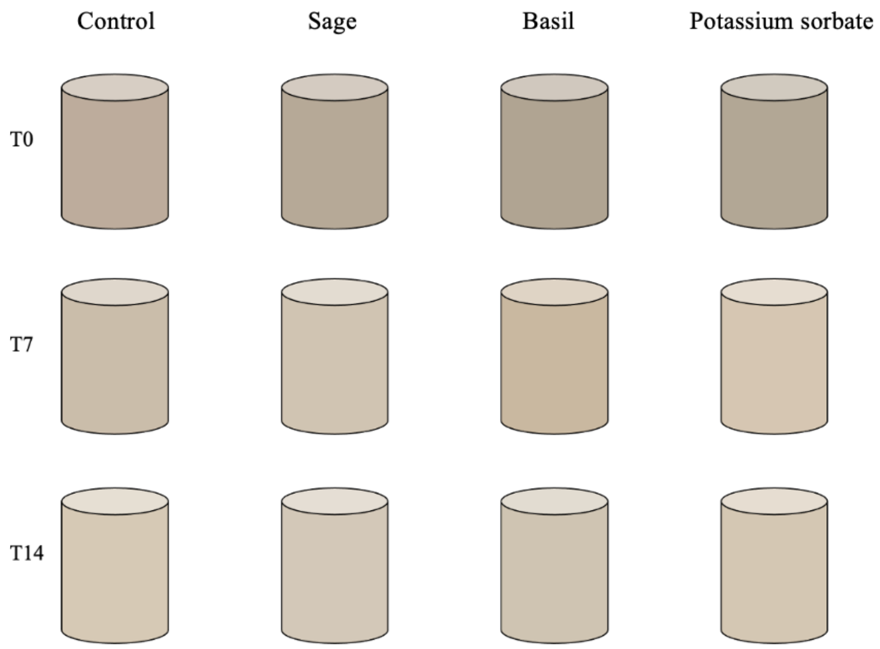

Figure S4. Color representation of the different yogurts over the 14 days with data collected from the colorimeter
